# Global phenotypic characterization of bacteria

**DOI:** 10.1111/j.1574-6976.2008.00149.x

**Published:** 2008-12-01

**Authors:** Barry R Bochner

**Affiliations:** Biolog Inc.Hayward, CA, USA

**Keywords:** phenotyping, phenomics, metabolomics, phenotypic analysis, cell phenotypes, phenotypic characterization

## Abstract

The measure of the quality of a systems biology model is how well it can reproduce and predict the behaviors of a biological system such as a microbial cell. In recent years, these models have been built up in layers, and each layer has been growing in sophistication and accuracy in parallel with a global data set to challenge and validate the models in predicting the content or activities of genes (genomics), proteins (proteomics), metabolites (metabolomics), and ultimately cell phenotypes (phenomics). This review focuses on the latter, the phenotypes of microbial cells. The development of Phenotype MicroArrays, which attempt to give a global view of cellular phenotypes, is described. In addition to their use in fleshing out and validating systems biology models, there are many other uses of this global phenotyping technology in basic and applied microbiology research, which are also described.

## Introduction

Phenotypes are observable characteristics of cells. In recent years, the term ‘phenotype’ has been applied broadly to include any cell property, including ‘molecular phenotypes’ such as the mRNA level of a single gene. Throughout this review, I will be referring to phenotypes in the more traditional sense of properties related to cell growth. Growth phenotypes define if and how fast a bacterium will grow. They have a particular advantage in that they can be easily observed, scored, and measured without requiring expensive technology. Furthermore, when cells lose their normal phenotypes due to mutations that result in poor-growth phenotypes, these can also be used to advantage in positive selections to obtain secondary mutants that restore better growth. Analysis of the range of these ‘suppressor’ mutations restoring good growth has been a proven method to accurately expand our knowledge of gene function, protein and pathway interactions, and microbial biology.

## The importance of growth phenotypes

Growth phenotypes allow microbiologists to describe and thereby differentiate cells. The power of phenotypic description of bacteria was demonstrated in a systematic way by the doctoral dissertation of L.E. den Dooren de Jong, working first under M.W. Beijerinck and completing the project under A.J. Kluyver at the Technological University at Delft, the Netherlands (den Dooren de Jong, 1926). He showed that bacteria could be readily distinguished by growth assays on agar media with several hundred C- and N-sources. In the same decade, with the publication of the first edition of the *Bergey's Manual of Determinative Bacteriology* in 1923, microbiologists began to systematically describe and define bacterial species based on lists of phenotypes, primarily growth related.

Evolutionary forces will drive bacteria to grow in as many environmental niches as possible. Species are formed as they are selected for survival and faster growth in various niches. This typically involves two fundamental aspects. First, bacteria evolve to use the basic elemental nutrients in that environment that are common to and essential for all growth: C, N, P, S, O, H, etc. Second, bacteria evolve to survive against potentially toxic aspects of an environment: toxic chemicals, temperature, pressure, electromagnetic radiation, desiccation, etc. Only about 9000 bacterial species have been described and named (http://www.bacterio.cict.fr/number.html– the number of named fungal species is about 10 times higher) but the actual number of species on Earth has been estimated to be in the range of 10^5^–10^9^. These large numbers are indicative of the diversity of ecological niches on our planet.

Therefore, growth phenotypes are directly and intimately involved in fundamental aspects of cellular genome and organism evolution and they remain a cornerstone of microbial taxonomy. Valid species definitions require phenotypic description (http://www.bacterio.cict.fr/).

## The need to detect phenotypes globally

There are many needs that drive the desire to detect phenotypes globally. One is in the area of systems biology, the topic of this special issue. Systems biologists are attempting to make computerized models that accurately mimic behaviors of a cell and, more importantly, to predict behaviors that may not be apparent. To test their models they need real data showing, with as much detail as possible, how components of cells change under ‘interesting’ experimental conditions. Biologists understand that the cell is a system, but we are just at the early stages of describing cells as such. Providing the data underpinning such efforts are relatively new tools for global cellular analysis.

The first global analytical tool developed was for proteins: the two-dimensional protein gel electrophoresis method described in 1975 (O'Farrell, 1975). This enabled the analysis, in a single experiment, of levels of most proteins in a cell. It has ultimately become the foundation of proteomics, the first of the current ‘omics’. Neidhardt and colleagues (Neidhardt *et al.*, 1983; VanBogelen *et al.*, 1999) were the first to pursue this technology and provide basic underpinnings of systems biology by attempting to identify and quantify each protein spot that could be detected from the bacterium *Escherichia coli*. This was only partially successful because many proteins could not be attributed at that time. Nevertheless, experiments could be performed where *E. coli* was cultured under different growth conditions, and changes could be clearly detected in the protein composition of the bacterium. Detecting proteins globally led to a greater understanding of how *E. coli* globally coordinated its protein content under different growth or stress conditions. In turn, this led to the observation and enumeration of groups of proteins regulated in a common way, the so-called ‘regulons’.

In the 1980s and 1990s, other global tools were developed. Sasser and colleagues (Kunitsky *et al.*, 2006) developed a biochemical analysis method for membrane lipids, Venter and colleagues (Fraser *et al.*, 1995) developed a method for sequencing entire genomes and Fodor *et al.* (1993) developed a method for measuring the mRNA levels in cells. These powerful biochemical and molecular methods have revolutionized biological research. Yet, with all the information that can be obtained with these tools, there is still not enough of the right kind of information to evaluate complex models and allow physiological and biological conclusions to be drawn. As described in this review, phenotypic information is a very useful form of information that complements the current information obtained from global biochemical and molecular analysis.

A second major need is in the area of microbial physiology. Physiology provides a useful and appropriate way to describe cells and differences between cells. It enumerates fundamental functions that a cell must perform, and then it describes, in whatever detail is appropriate, the specifics of how a cell achieves each function. Examples of fundamental physiological processes in microbial cells are maintenance of water activity, internal pH, energy production, DNA replication, cell division, biosynthesis of all needed biochemical components, and sourcing the C, N, P, S, O, H, etc. that a cell needs to accomplish all of these activities. Throughout this review, more detail and examples are provided to illustrate how global phenotypic analysis helps address the need for a better understanding of microbial physiology.

A third major need is in the area of microbial taxonomy. As indicated in the previous section, microbial species require phenotypic descriptions. With the advent of rapid DNA sequencing, new species are being discovered by both 16S rRNA gene sequencing and sequencing of metagenomes. The DNA evidence of the existence of thousands of novel species is clear, but parallel advances in cell culture and phenotyping are needed to actually describe the biology of these bacteria and work with them experimentally. Furthermore, it would be highly desirable to standardize the phenotypic descriptions of bacteria. Just as all bacteria can be described with a powerful common framework of their 16S rRNA gene or genomic DNA sequences, it would also be highly desirable and productive to describe all bacteria by their phenotypes, which reflects their physiology. The current status of phenotypic descriptions is unacceptable. In the major taxonomic publications such as *Bergey's Manual* and *The Prokaryotes*, groups of microorganisms are assigned to review by various experts, who describe the cells with a nonuniform set of phenotypes, based on historical practices and practical technical limitations. Instead, a more ideal approach would be to convene a meeting of microbial physiologists and taxonomists charged with the task of agreeing upon an extensive standard list of phenotypes that could be used as a common set to categorize all microbial species. Then what is needed is a practical technology to analyze all microbial species and provide a database of these standard phenotypes.

In addition to these three major needs, there are a number of practical and important uses of global phenotyping that are described subsequently in this review.

## How to accomplish the goal

**Fig. 1:**
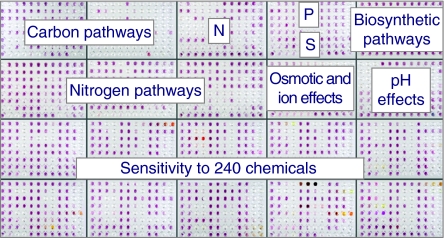
The 1920 phenotypic assays in the PM set for bacteria. PMs are sets of phenotypic assays performed in 96-well microplates. The microplate wells contain chemicals dried on the bottom to create unique culture conditions after rehydration. Assays are initiated by inoculating all wells with cell suspensions. After incubation, some of the wells turn various shades of purple due to reduction of a tetrazolium dye as the cells respire. The variable level of purple color indicates that the cells are metabolically active and respiring in some wells but not others. Other colors such as orange are the colors of other chemicals in the wells. Microplates in the PM set are organized into functional groups as labeled in the Figure. Assays of C, N, P, and S metabolism provide information about which metabolic pathways are present and active in the cells. Assays of ion, pH, and chemical sensitivities provide information on stress and repair pathways that are present and active in cells.

In the past, phenotypes have been measured one at a time. What was needed was a way to measure hundreds or thousands of phenotypes at a time. The method for accomplishing this would ideally work for all cells and be as simple and inexpensive as possible. With these objectives in mind, my colleagues and I set out to develop a method for global analysis of cell phenotypes, which we called ‘Phenotype MicroArrays’ (PMs). We developed a standard set of nearly 2000 assays that could be used productively with a very wide range of bacterial species (Bochner *et al.*, 2001; Bochner, 2003). The assays are performed in 100-μL cultures in 96-well culture plates. As shown in Fig. 1, the set consists of about 200 assays of C-source metabolism, 400 assays of N-source metabolism, 100 assays of P-source and S-source metabolism, 100 assays of biosynthetic pathways, 100 assays of ion effects and osmolarity, 100 assays of pH effects and pH control with deaminases and decarboxylases, and 1000 assays of chemical sensitivity. In the chemical sensitivity assays, there are 240 diverse chemicals, each at four concentrations. We have attempted to select chemicals that are toxic to most microorganisms and that are toxic by interfering with diverse cellular pathways, for example, DNA replication, RNA transcription, protein synthesis, cell wall synthesis, cell membrane synthesis, nutrient transport, etc. For purposes of including chemicals found in natural environments, we have also incorporated into this set, tests to measure the sensitivity of bacteria to numerous inorganic chemicals, such as cations (Na^+^, K^+^, Fe^3+^, Cu^2+^, Co^2+^, Zn^2+^, Mn^2+^, etc.) and anions (chloride, sulfate, chromate, phosphate, vanadate, nitrate, nitrite, selenite, tellurite, etc.).

The PM consists of two components, which are combined to start the assay. First is a suspension of cells. The bacterial cells are typically pregrown on a nearly universal agar medium for copiotrophic bacteria, Biolog Universal Growth Agar with Blood (BUG+B; however, other media can also be used – e.g. R2A agar is a good general medium to use for oligotrophic bacteria) and a cotton swab is used to prepare a cell suspension at a standardized cell density. The cell suspension has some salts to maintain cell viability, and a tetrazolium redox dye chemistry to measure cell respiration. This inoculum is simply pipetted into the wells of the 20 microplates (PM1–20). The microplate wells have, dried down on the bottom, the other nutrients and/or chemicals needed to create the 1920 unique culture conditions for the PM assay set.

**Fig. 2:**
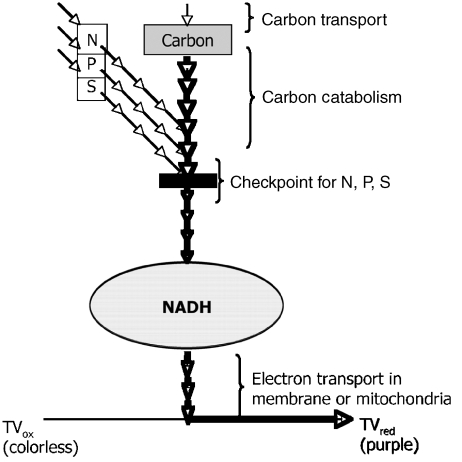
The coordinated linkage of metabolic pathways. Schematic diagram of major metabolic pathways in bacteria and how their activities are converted to a colorimetric readout. A C-source that can be transported into a cell and metabolized to produce NADH will engender a redox potential and flow of electrons to reduce a tetrazolium dye (Bochner & Savageau, 1977) such as tetrazolium violet (TV), thereby producing purple color. The more rapid this metabolic flow, the more quickly purple color is formed. However many cells exhibit a phenomenon of checkpoint control, where the catabolism of the C-source is restricted if the cell does not also have sufficient levels of N, P, and S. This enables assays where one can also measure these N, P, and S catabolic pathways. The more active they are, the more rapid the catabolism of the C-source and the more quickly purple color is formed.

**Fig. 3:**
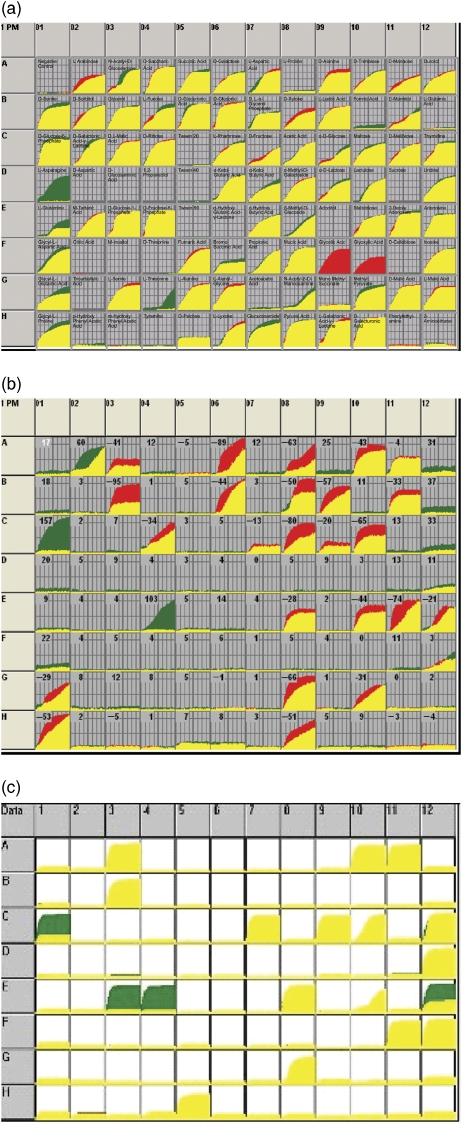
Using PM technology to detect changes in C metabolism. (a) An example of the compared C-source metabolic activities of two bacterial strains. Assays were performed in Biolog PM1 MicroPlates that contain a negative control well (well A1) and 95 different potential C-sources. Kinetic data were collected using the Biolog omnilog instrument and software. The curves show the time course (horizontal axis) of the amount of purple color formed from tetrazolium dye reduction (vertical axis) in each of the 96 wells. Data from one strain are shown in red, the other strain in green, and yellow is the overlapping of the two kinetic curves. C-sources more rapidly metabolized by the first strain are shown in red (F9, glycolic acid; F10, glyoxylic acid), and by the second strain are shown in green (D1, l-asparagine; G4, l-threonine), and metabolized equally are shown in yellow. (b) An example of the compared C-source metabolic activities of a strain of *Yersinia pseudotuberculosis* strain 15 478 when tested at two temperatures. The strain was assayed for C-metabolism using Biolog PM1 MicroPlates. For most C-sources, metabolism was more rapid at the warmer temperature. However, compared with the warmer temperature of 33°C (shown in red) the strain showed increased metabolism of three C-sources at 26°C (shown in green: well A2, l-arabinose; C1, d-glucose-6-PO_4_; E4, d-fructose-6-PO_4_). (c) An example of the compared C-source metabolic activities of an isogenic pair of *Listeria monocytogenes* strains (P14 vs. P14 prfA^*^). The strains were assayed for their C- metabolism using Biolog PM1 MicroPlates. Compared with its wild-type parental strain (shown in red), the hyperpathogenic prfA^*^ strain (shown in green) exhibited increased metabolism of hexose phosphates as C-sources (well C1, d-glucose-6-PO_4_; E3, d-glucose-1-PO_4_; E4, d-fructose-6-PO_4_).

The basis for these assays is a nearly universal culture medium that contains all the nutrients needed for cell growth (e.g. C, N, P, S, K, Na, Mg, Ca, Fe, amino acids, purines, pyrimidines, and vitamins). To measure which C catabolic pathways are active in a bacterium, the wells in PM1 and 2 contain this medium with all ingredients provided at sufficient levels except the C-source, which is omitted. The various wells in PM1 and 2 provide 190 alternative C-sources. For any of these C compounds, if the cell has a transport system and a catabolic pathway for that chemical, it will catabolize the chemical, producing NADH in the process. The electron transport pathway in the cell will then take electrons from NADH and pass some of them on to the tetrazolium dye as shown in Fig. 2. Reduction of the tetrazolium dye due to increased cell respiration results in formation of a purple color in the well. C-sources that are strongly metabolized rapidly form a dark purple color, whereas C-sources that are weakly metabolized slowly form a light purple color (Fig. 1). The rates and extent of color formation in each well of the PM can be monitored and recorded by the omnilog instrument (Bochner, 2003), which is essentially an incubator holding 50 microplates, with a color video camera interfaced to a computer. The outputs of the omnilog are color-coded kinetic graphs. When two strains are compared, one is shown in red, another in green, and the overlap in yellow. Hence yellow indicates no change in phenotype and the red or green color indicates more rapid metabolism by that strain (see Fig. 3).

Similarly, to measure which N catabolic pathways are active in a bacterium, the wells in PM3, 6, 7, and 8 contain the medium with all nutritional ingredients provided at sufficient levels except that the N-source is omitted. The various wells in these panels provide 380 alternative N-sources. In the case of N-supplying pathways (and also P- and S-supplying pathways in PM4 and other nutrient-supplying pathways in PM5), these are measured indirectly by their linkage to C/energy metabolism. In many, but perhaps not all bacteria, cells coordinate their primary nutritional pathways. If the cell cannot grow due to another limitation, its control mechanisms restrict the unproductive transport and metabolism of C-sources. For these bacteria, if they are starved for N, P, S, or some other essential nutrient (e.g. an amino acid, nucleoside, vitamin, etc.), then they do not catabolize C. This coordinated linkage of C/energy catabolism is shown in Fig. 2 as a ‘checkpoint’. Catabolism of C is arrested unless N, P, S, and essential nutrients are also provided. Linkage of metabolism is also the basis for detecting N, P, S, and biosynthetic pathways using the same tetrazolium redox assay used to measure C metabolism. I am not aware of this phenomenon being described previously, but we clearly detect it in hundreds of bacterial species with our PM assay.

How is this linkage or ‘crosstalk’ mediated at a molecular level? Without direct evidence one can only speculate. In the case of amino acid starvation, it could be mediated by the alarmone ppGpp (Stephens *et al.*, 1975), which is formed when the ribosome is stalled. However, a more simple and direct possibility is that the cell is sensing that it is becoming too reduced. When it has a good supply of a catabolizable C-source but it cannot grow due to another deficiency, there is no consumptive outlet for its energy production. NADH levels are high and consequently NADPH levels also rise, because NADPH is not being utilized for biosynthesis. This imbalance in excessive NADH and NADPH would be a logical molecular alarm signal to indicate to the cell that it is producing more energy than it can use and thereby provide the ‘checkpoint signal’ to stop unneeded and potentially harmful generation of excessive reducing power.

**Fig. 4:**
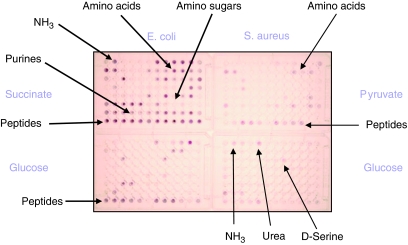
Regulation of N metabolism by C metabolism is different in *Escherichia coli* vs. *Staphylococcus aureus. Escherichia coli* and *S. aureus* change their N-source metabolism in very different ways in response to the C-source they have available. Assays were performed in Biolog PM3 MicroPlates, which contain a negative control well (well A1) and 95 different potential N-sources. Cells are inoculated in a suspending medium with different C-sources, such as glucose, pyruvate, and succinate, as indicated in the figure. In *E. coli*, glucose represses the activity of many N-catabolic pathways and the set of active pathways is a subset of the pathways active on succinate. By contrast, in *S. aureus*, glucose represses the activity of many N-catabolic pathways but it also activates an entirely different set of active pathways compared with pyruvate.

PM technology can also be used to directly observe metabolic crosstalk between different areas of metabolism. An illustration of this is shown in Fig. 4. It is well known in *E. coli* that C metabolism regulates N metabolism (Maheswaran & Forchhammer, 2003). When a preferred C-source is present, such as glucose, the transport and metabolism of many N-sources is restricted or shut off. Conversely, when a poor C-source is provided, such as succinate, the transport and metabolism of virtually all of *E. coli*'s numerous N-sources is turned on. In *E. coli*, this is mediated in part by cyclic AMP levels, but the interactions and intricacies of C and N metabolism have not been studied systematically in diverse microbial species.

Certainly, metabolic regulation is different in other bacterial genera. Genera such as *Pseudomonas, Acinetobacter*, and *Achromobacter* do not have the same preference for sugars as C-sources, and instead may prefer amino acids, carboxylic acids, and/or fatty acids. In these genera, the hierarchy may be reversed or rearranged in some interesting way. A striking example of different regulation is shown by the comparison of *E. coli* to *Staphylococcus aureus* in Fig. 4. Whereas *E. coli* exhibits a hierarchical form of crosspathway regulation with fewer N-sources utilized with glucose, as compared with succinate, *S. aureus* uses an entirely different set of N-sources with glucose, as compared with pyruvate. Much work remains to be carried out to fully describe this important crosstalk regulation in various microbial genera.

## Advantages of measuring respiration instead of growth

As described, PMs measure cellular phenotypes colorimetrically using a tetrazolium redox dye to measure cell respiration. Why measure respiration instead of growth? There are at least three reasons. First, it is a more sensitive way to measure phenotypes. Cells may respond metabolically by respiring but not growing. For example, *Staphylococcus* species give a respiratory response to N-sources but we have never been able to get them to grow on single N-sources. Second, it allows the measurement of more cellular pathways. For example, *E. coli* has a formate dehydrogenase pathway that can be detected by respiration but not by growth. It can respire and generate energy with formate, but it cannot grow on formate because it is not able to convert this one-carbon compound into the larger molecules that it needs in order to replicate. Third, it can be used to measure phenotypes of cells that cannot be cultured axenically, for example *Coxiella* species that must be cultured inside of eukaryotic cells. Omsland and Heinzen (Bochner *et al.*, 2008) have recently reported success in using information from PM assays on *Coxiella burnetii* that were recovered after mechanical disruption from host mammalian cells. This information on nutrients that stimulated or inhibited respiration was used to develop a complex *Coxiella* medium, and Omsland is now able to keep these bacteria metabolically active in a cell-free environment, for >24 h. This allows him to use pulse labeling with radioactive isotopes to study *de novo* protein synthesis in a cell-free environment.

There are also some complications and limitations to PMs that should be mentioned, especially if the microorganism is very slow growing or requires more extreme culture conditions. With prolonged incubation, especially at higher temperatures, the wells can dry out. This problem can be handled by sealing the microplates with a clear plastic tape or by incubating them in a plastic bag or with high humidity. At warmer temperatures, abiotic dye reduction reactions can be catalyzed, and at temperatures exceeding 80 °C the microplate plastic starts to melt. For microorganisms requiring special gas atmospheres, the microplates must be incubated inside of plastic bags with low gas permeability. Under high-salt conditions, the nutrients and dye chemistry in the well can partially precipitate, resulting in weaker color formation. Alkaline pH conditions make the redox chemistry more easily reduced and may catalyze abiotic reduction, whereas acidic pH conditions may inhibit or completely block dye reduction, or shift the spectral properties of the redox dye from purple toward red.

## Uses in studying gene function

Systems biologists would typically view gene function as a descriptive unit of genome annotation, and ideally such annotation would fully describe all functions of all genes. Current annotation, however, is incomplete and often based on extrapolation that is not verified and may be incorrect. As biologists move toward the enormously challenging goal of fully understanding the function and role of all genes in all cells, a direct way to assay gene function is to examine cells with knockouts of genes and see if accurate predictions can be made of how the loss of that gene will affect the phenotypes of the cell. This has, in fact, been the largest single use of PM technology.

Genetic tools now allow genes to be knocked out by a variety of methods. The first large undertaking applying PMs to gene function was an attempt to try to define the function of all genes in *E. coli* encoding two-component regulatory systems. This work (Zhou *et al.*, 2003) took advantage of a method recently introduced for making precise deletions of the entire gene (Datsenko & Wanner, 2000). To be certain that the deletion mutants were constructed correctly, in addition to sequencing the relevant genomic region of each mutant, two mutant clones were picked and assayed for phenotypic changes with PM. Surprisingly, and as a note of caution, in a significant fraction of these (about 10%), the two mutant clones exhibited different biological changes, indicating that other genetic changes were inadvertently introduced. A third mutant was tested in these cases to resolve which mutants were the true single-gene deletions. This observation further demonstrates the power of PM technology to detect small differences between closely related strains and to verify the accuracy of genetically modified microorganisms. Other laboratories have also performed studies that reinforce this point. Miller and colleagues (Funchain *et al.*, 2000) tested mutator strains of *E. coli* and found that they could use PMs to readily detect phenotypic changes affecting C metabolism in 70% of strains after about 1000 generations of growth. Eisenstark and colleagues (Tracy *et al.*, 2002) examined stains of *Salmonella* that had been stored in soft agar stabs at room temperature for >40 years. By testing C and N metabolism phenotypes with PM1 and PM3 plates, they found that many strains had lost metabolic functions and some had actually gained metabolic functions, presumably due to genetic alterations.

Numerous published examples document the successful use of PM technology to assay phenotypic changes in gene knockout strains using a wide range of microbial species (http://www.biolog.com/mID_section_13.html). Many of the gene knockout assays have been of regulatory genes (Pruess *et al.*, 2003; Kapatral *et al.*, 2004; Shakarji *et al.*, 2006; Jones *et al.*, 2007; Li & Lu, 2007; Mascher *et al.*, 2007; Bailey *et al.*, 2008; Perkins & Nicholson, 2008; Zhang & Rainey, 2008), but some have looked at genes coding for enzymes (Koo *et al.*, 2004; Van Dyk *et al.*, 2004; Biswas & Biswas, 2005; Lee *et al.*, 2005, 2007; von Eiff *et al.*, 2006; Chen *et al.*, 2007; Bailey *et al.*, 2008) or genes of unknown or poorly documented function (Chouikha *et al.*, 2006; Erol *et al.*, 2006; Loh *et al.*, 2006). A nice illustration in which the function of an entire operon was annotated is the work of Kustu and colleagues (Loh *et al.*, 2006) where they discovered the existence of a novel pathway for breakdown of pyrimidines by *E. coli*.

There are certainly gene families in cells and evidence of genes that are either partially or fully redundant. To determine the function of these genes, it is often necessary to knock out multiple genes. For example, Nishino and colleagues (Bochner *et al.*, 2008) examined knockout mutants of nine putative efflux pumps in *Salmonella* and discovered unanticipated connections between efflux pumps, metal metabolism, and pathogenicity. The largest gene knockout project so far has been undertaken by Hirotada Mori's group (Ishii *et al.*, 2007; Baba *et al.*, 2008; Tohsato & Mori, 2008), which has made knockouts of all essential genes of *E. coli* MG1655 and assayed 500 knockout strains with PM. In addition to PM analysis, the single-gene knockouts were screened for growth on minimal medium and it was found that nearly all could still grow, indicating the availability of alternative metabolic pathways to compensate partially if not fully. Work is now in progress to make double-gene knockouts to provide additional phenotypic information for testing systems biology metabolic models (Bochner *et al.*, 2008).

The one systematic attempt to use PM technology to assess the accuracy of current genome annotation is the work of Paulsen and colleagues (Johnson *et al.*, 2008). They analyzed knockout mutants of 78 presumptive transporter genes of *Pseudomonas aeruginosa* to see which phenotypes were altered. Twenty-seven of the 78 knockouts gave clear phenotypes, and of these only 12 (44%) precisely matched the predicted annotation. In 10 (37%), a more precise annotation was obtained, and in five (18%), a significant reannotation was enabled. New transporters were found for hydroxy-l-proline, *N*-acetyl-l-glutamate, and histamine. This is the first time a histamine transporter has been annotated, and its discovery could assist work in studies of histamine's role in the human immune system.

## Uses in studying pathogenicity and epidemiology

There are a number of laboratories using PM technology in novel ways to delve into various aspects of bacterial pathogenicity and the related issue of epidemiology. Diverse pathogens have been analyzed, including *E. coli, Salmonella enterica, P. aeruginosa* and *Pseudomonas syringae, Enterobacter* (now *Cronobacter*) *sakazakii, Yersinia pestis, Vibrio cholerae, Campylobacter jejuni, Helicobacter pylori, S. aureus, Listeria monocytogenes, Mycobacterium* sp., *C. burnetii*, and *Legionella pneumophila*.

One of the most interesting and productive series of works has come from studies of the highly clonal pathogen, *S. enterica* serovar Enteritidis. Different strains of this pathogen share 99.99% genomic identity; yet, surprisingly, they vary greatly in their pathogenic properties. Guard-Bouldin and colleagues used PM technology to compare two strains of the same phage type (PT13a), with clearly distinct biological properties: one is a biofilm-forming strain and a good colonizer of chickens but does not infect eggs, whereas the second does not form biofilms but does infect eggs. Coinfection with both subtypes causes the most serious infections and disease spread (Guard-Bouldin *et al.*, 2004). In spite of the very close genomic relatedness of these strains and the inability of genetic typing methods (DNA microarray hybridization, pulsed-field gel electrophoresis, and ribotyping) to detect significant polymorphisms (Morales *et al.*, 2005, 2006), PM technology quickly uncovered many phenotypic differences between these two strains (the egg-infecting strain is more metabolically active) and provided essential information that has led to the elucidation of 447 small-scale genetic polymorphisms that appear to be important hot spots for genetic change such as the d-serine operon.

Interestingly, the d-serine operon was found to be a hot spot for genetic alteration in another highly clonal pathogen, *E. coli* O157. By analyzing a large collection of strains from foodborne outbreaks, Cebula and colleagues (Mukherjee *et al.*, 2006) first found a sucrose-positive, d-serine-negative phenotype common to most O157 strains and subsequently confirmed that a sucrose operon had inserted into the d-serine operon. Genetic analysis of this genome region showed that it was a hot spot for genetic mosaicism. Cebula's group concluded that phenotypic analysis is a very useful tool in strain attribution (Bochner *et al.*, 2008). In a comparative PM analysis of the O157 strain from the summer, 2006, spinach outbreak in the United States, they showed (Mukherjee *et al.*, 2008) that it had a rare *N*-acetyl-d-galactosamine-negative phenotype, which had only been found once previously. Both the Cebula and Guard-Bouldin laboratories have shown that, especially with clonal pathogens, it can be easier, more efficient, and more productive to go from phenotype back to genotype, instead of starting with genomic analyses. One other approach demonstrating the usefulness of analyzing phenotypes of natural isolates is the work of Hutkins and colleagues (Durso *et al.*, 2004), where PM analysis of C metabolism showed significant differences between commensal strains of *E. coli* from cattle vs. O157:H7 strains. These differences in metabolic capabilities are likely to contribute to colonization capabilities in various environments.

An area of particular interest is the use of PM technology to examine changes in the physiology of a bacterium peculiar to stages of pathogenic adaptation *in vivo*. Preston and colleagues (Rico & Preston, 2008) analyzed phenotypic changes of the plant pathogen *P. syringae*, growing in laboratory culture media vs. growing in one of its environmental media – tomato apoplastic fluid. As previously mentioned, Omsland and Heinzen (Bochner *et al.*, 2008) investigated the metabolic phenotypic properties of *C. burnetii* extracted from culture inside of mammalian cells. In a work on a related pathogen, *L. pneumophila*, which can also survive and grow in macrophages by evading the endocytic-lysosomal destruction pathway, Swanson and colleagues produced two novel and exciting findings. First (Sauer *et al.*, 2005), they used PMs to show that *phtA* (phagosomal transporter defective) mutants require l-threonine for replication and that this amino acid triggers differentiation of the cell from a motile transmissive form to the nonmotile replicative form. More recently (Dalebroux *et al.*, 2008; Edwards *et al.*, 2008), this group reported using PM technology to screen a flaA–gfp fusion strain (an indicator of differentiation back to the motile transmissive state) under hundreds of culture conditions and discovered that growth arrest and the transition to the motile transmissive form is triggered by carboxylic acids, especially short-chain fatty acids.

## Environment sensing and pathogenesis – the importance of metabolic and temperature signals

Changes in culture conditions can trigger changes in pathogenic bacteria in interesting and important ways (Mekalanos, 1992) and temperature seems to be one of the clearest and most relevant examples of this. The normal temperature of humans and many mammals is 37 °C, which is toward or above the upper limit of temperatures tolerated by most fast-growing environmental bacteria and fungi. Febrile temperature elevation in response to microbial infection extends the body temperature to even higher levels. It is clear that at least some important human pathogens sense warm temperatures and turn on pathogenic functions in response.

Probably the best-documented example is *L. monocytogenes*, which is notable for its ability to grow at low temperatures on foods stored in refrigerators. In this bacterium, a large set of pathogenic genes are regulated by the *prfA* gene, and the PrfA protein coded for by this gene is temperature induced (Vazquez-Boland *et al.*, 2001; Scortti *et al.*, 2007). Other pathogens in which we have seen dramatic phenotypic changes in response to temperature include *Bordetella parapertusis, Yersinia pseudotuberculosis*, and *Y. pestis*, which also changes phenotypes in response to Ca concentrations (Holtz *et al.*, 2004). An example of temperature effects on C metabolism of *Y. pseudotuberculosis* is shown in Fig. 3b.

Temperature is a potential signal to inform a pathogen that it is inside of a warm-blooded animal, but what are the signals that inform cells that they are inside of animal cells, or inside of specific cellular organelles? Macromolecular structures such as specific proteins or membrane-surface structures of animal cells can be used as signals or binding sites, but what small molecules or physical/chemical signals might pathogenic bacteria also be sensing?

One important chemical signal is low Mg (Garcia *et al.*, 1996) and another could be high Ca (Mekalanos, 1992). Yet another important candidate for indicating a cytoplasmic location is hexose phosphates. Chemicals such as glucose-1-PO_4_, glucose-6-PO_4_, and fructose-6-PO_4_ would not be found in many environments, but would be in high concentrations in the cytoplasm of cells. Most cells take up sugars and in doing so the sugars are usually phosphorylated as part of the transport process. Therefore the intracellular environment presumably has most sugars in the form of sugar phosphates. My research group first noticed the importance of hexose phosphates independently as well as in collaboration with Vazquez-Boland. PM technology was used to test their pair of *L. monocytogenes* strains, one with a normal *prfA* gene and a second isogenic, hyperpathogenic strain with a constitutively induced prfA^*^ allele (Ripio *et al.*, 1997; Vega *et al.*, 2004). The most substantial phenotypic change we observed in the hyperpathogenic strains was increased metabolism of hexose phosphates as a C-source for growth (see Fig. 3c) in agreement with earlier findings from the Vazquez-Boland laboratory. This supports the idea that pathogenic *Listeria* are using hexose phosphates as a principle C-source for growth in the intracellular environment. In fact, Vazquez-Boland and colleagues have found that it may be the essential C-source. Uptake of the hexose phosphates in *Listeria* is mediated by the *hpt* gene, which is induced by the PrfA protein. If the *hpt* gene is deleted, *L. monocytogenes* is no longer pathogenic (Chico-Calero *et al.*, 2002). Even before this, our research group (Bochner, 1992) as well as Vazquez-Boland's group (Ripio *et al.*, 1997) independently discovered that metabolism of hexose phosphates for C and energy was a phenotypic property unique to the pathogenic *Listeria* species: *monocytogenes* and *ivanovii*. Adding additional support to the concept of signaling by hexose phosphates is the recent observation from Portnoy's laboratory (Schnupf *et al.*, 2006) that listeriolysin O toxin production by *L. monocytogenes* can be triggered by simply culturing the bacteria in laboratory media and adding glucose-1-PO_4_. A recent publication (Munoz-Elias & McKinney, 2006) more broadly reviews the data on the intracellular growth and C metabolism by bacteria.

As part of the pathogenic process, bacteria are often attacked and killed by cells of the immune system such as phagocytes. There are two cases where phenotypic test panels have shown dramatic changes in phenotype in response to the presence of serum: *Francisella tularensis* (Lifland, 1997) and *H. pylori* (Lei & Bochner, 2008). In both cases, the cells greatly increase the range of C catabolic pathways that are active when serum is present. We have speculated that this could be part of a sensing and defense mechanism for pathogens such as *Helicobacter* to sense bleeding as a precursor to attack by phagocytes. Subsequently, these invasive bacteria can be endocytically engulfed in lysosomal vesicles within the phagocytes in an acidic pH environment. Bacteria that can induce activities to tolerate the acidic conditions in lysosomal vesicles (as well as in the human stomach) can survive and cause persistent infections. Also, plant pathogenic bacteria have to sense and deal with acidic environments to grow in acidic plant apoplastic fluid (Llama-Palacios *et al.*, 2005; Rico & Preston, 2008). Therefore, an acid environment (e.g. pH 4–5) is expected to be another important signal for pathogenic bacteria to sense. In apparent agreement with this expectation, Datta *et al.* (2008) have recently examined a large collection of *L. monocytogenes* strains from different outbreaks using PM technology and found that strains involved in invasive listeriosis outbreaks seem to be more acid tolerant. However, strains involved in gastroenteritis outbreaks seem to be more osmotolerant.

Phenotype MicroArray test panels and protocols are now also available for mammalian cells, which opens up new potential avenues of studying the interaction of these cells as the pathogenic bacterium survives inside or alongside its host cell. It should be possible, by comparing the phenotypes of uninfected vs. infected mammalian cells, to gain information about how infection with a microorganism alters the biology of the host cell. The ultimate goal would be to measure, simultaneously and in real time, the phenotypes of both the bacterium and its host cell during a time course of infection. This is conceptually possible to achieve using two redox dyes of different redox potentials and colors, the higher redox potential dye for the bacteria and the lower redox potential dye for the mammalian cells. It remains to be seen if this can be done. The experiments performed thus far just scratch the surface of ways in which PMs can be used to examine pathogenesis, environmental signaling, and intracellular growth regulation.

## Uses in studying cell transformations – microscopic and macroscopic morphology

Cells are multistate automata. They constantly sense many aspects of their environment and try to adapt in the most advantageous way by reconstructing themselves. Even small changes in a cell's environment leads to changes in gene expression, protein levels, alterations in cellular organelles, walls, membranes, motility, and ability to form biofilms.

An important and unique aspect of PMs is that they allow the microbiologist to see, in one experiment, many faces of the cell being studied. PMs were designed from a physiological perspective to provide a diverse range of culture conditions including many different types of stress conditions such as insufficient C, N, P, S, and trace metals, osmotic, pH, and redox stresses of various types, inhibition of DNA, RNA, protein, cell wall and membrane synthesis, and exposure to naturally occurring and manmade toxic chemicals. By inoculating a microbial strain into the 1920 wells of a PM and incubating for a sufficient period of time, one can quickly and easily create 1920 different versions of their cell of interest.

This can be used productively in basic and applied research and development projects. For example, Bohm and colleagues (Bochner *et al.*, 2008) used PMs to screen for and find culture conditions that trigger biofilm formation in *E. coli*. They made the surprising observation that sublethal concentrations of translation-inhibiting antibiotics (tetracyclines, aminoglycoside, amphenicols, and macrolides) were effective biofilm-triggering agents. Wenyuan Shi and colleagues (Chavira *et al.*, 2007) used PMs to screen for and find culture conditions that altered the morphology of fruiting bodies in *Myxococcus xanthus*. Many microorganisms have genes coding for pathways of secondary metabolism and these pathways are often turned on only under a specific set of stress conditions. For example, antibiotic production by actinomycetes (Bibb, 2005) and toxin production by toxigenic bacteria (Mekalanos, 1992) are regulated in ways that are not well defined and understood. PMs can be used as a tool for efficiently testing a cell to help define the culture conditions and stresses that turn on a pathway of secondary metabolism.

Culture conditions are also responsible for triggering morphological development in microorganisms, such as filamentation, germination, and sporulation. The important fungal human pathogen, *Candida albicans*, undergoes a transition from a single-celled yeast form to a pathogenic and invasive filamentous form when exposed to special culture conditions of nutrition, pH, and temperature (Gale *et al.*, 1998; Liu, 2001; Haoping, 2001; Hauser *et al.*, 2002). Irina Druzhinina and colleagues (Friedl *et al.*, 2008a,b) performed a series of elegant experiments to study sporulation in the mycoparasitic and cellulose-degrading fungus *Trichoderma atroviride*. In the first photobiology application of PM technology, they tested the hypothesis that photostimulation is the primary inducer of sporulation. PM plates were exposed to light, thereby superimposing conditions of incident light with other culture and stress conditions. They found that, contrary to accepted beliefs, light plays a much lesser role in conidiation by *T. atroviride* than C-source, which is the dominant determining factor. Furthermore, these studies revealed a crosstalk between effects of metabolism of cyclic AMP, illumination, and response to oxidative stress.

## Uses in understanding the diverse biology of bacteria – general phenotypic and culture properties

Many microbial cells remain ‘unculturable’ or very difficult and slow to culture. When these cells are important pathogens, the inability to culture them slows the progress in studying them and hinders the entire process of finding curative treatments. PM technology provides a set of nearly 2000 culture conditions under which one can test the ability of a microorganism to respire and grow. This set includes hundreds of C-sources, N-sources, P- and S-sources, nutrient supplements, and various conditions varying the pH, ion, and osmotic status of the culture environment. By inoculating and incubating PM panels with a microorganism that can be cultured in an undefined medium, one can systematically test what stimulates growth, and equally important, what inhibits growth. The technology was first developed for *E. coli* (Bochner *et al.*, 2001; Zhou *et al.*, 2003) but now there are protocols available to permit the testing of >1000 bacterial species as well as most yeast and filamentous fungi.

In a previous paragraph, we have already cited the example of the work of Omsland and Heinzen to culture *C. burnetii*, which has been, until now, considered an obligate intracellular parasite. Using PM technology to determine nutrients that stimulated respiration, they have ultimately succeeded in optimizing axenic culture conditions so that it can be maintained in a viable and metabolically active state outside of its host cell for >24 h (Bochner *et al.*, 2008). This has been made possible because PMs can read out even a slight stimulation of respiration when cells are still not growing. Xiang-He Lei in our laboratory tested a strain of *Halobacterium salinarum* provided by Schmid and Baliga (Schmid *et al.*, 2007). This bacterium is an extreme halophile, preferring to grow in a NaCl concentration of 25%, which causes precipitation problems and makes it challenging to measure cell respiration colorimetrically with dyes. Nevertheless, Lei was able to determine that its preferred C, N, and P nutrients were glycerol, glutamine, and glycerol-PO_4_. The preference for glycerol makes sense ecologically as this chemical is an effective osmotic balancer and it is found abundantly in high-salt environments. Lei also made the surprising and interesting observation that inorganic phosphate not only is not a preferred source of P but it is actually toxic to *Halobacterium* at low millimolar levels.

A final and perhaps most important (in terms of its widespread infectivity in humans) example is the mycobacteria. Some species of *Mycobacterium* grow rapidly but many grow slowly or extremely slowly, for example, *Mycobacterium bovis, Mycobacterium avium*, and even more so *Mycobacterium tuberculosis* and *Mycobacterium leprae. Mycobacterium bovis* strains have a doubling time of about 24 h. They take about 2 weeks to culture in liquid and 4–8 weeks to form colonies on the most optimal agar culture media, and as with other mycobacteria, their metabolic properties have remained refractory to study. Wheeler and colleagues (Upadhyay *et al.*, 2008) recently reported the successful development of a PM protocol for testing strains of *M. bovis*, in spite of having difficulties in consistently eliminating levels of background metabolism. They compared the Pasteur and Russian strains of *M. bovis* BCG, which have a long and important history in medical microbiology as general immunological adjuvants, and as attenuated vaccine strains for tuberculosis. The Pasteur strain is a low producer of the key antigen protein mpb70 whereas the Russian strain is a high producer. PM analysis that required only 5 days (because it measures respiration instead of growth), indicated that both strains could utilize glucose, pyruvate, glycerol, dihydroxyacetone, Tweens, and methyl-succinate, but there were also several differences that distinguished the strains. Differences were reported (Bochner *et al.*, 2008) in both their C metabolism (for d-lactose, cellobiose, gentiobiose, amygdalin, salicin, l-asparagine, d-alanine, l-alanyl-glycine, fumaric acid, and bromo-succinic acid) and their N metabolism (l-glutamine). These phenotypic assays provide not only the first detailed metabolic strain characterizations, but they may lead to more rapid diagnostic capabilities as well as better methods for culturing and isolating mycobacteria.

## Uses in taxonomy, bacterial identification, microbial ecology, and evolution

The existence of so many bacterial species reflects the presence of so many environmental niches on Earth and the resourcefulness of life forms to evolve to live in them. This topic was already introduced in a previous section along with a perspective on the historical role of phenotypic assays in microbial characterization and species description. A major advantage of 16S rRNA gene sequences in microbial taxonomy is that it is universally applicable and taxonomically predictive. Phenotypic testing is also universally applicable (to culturable microorganisms) and taxonomically predictive, and in addition it provides useful information about the biological properties of cells. However, before PM technology, there was not a sufficient number of phenotypic tests, and the same set of tests could not be used across a wide range of microbial species.

Now there are protocols, using the same set of 1920 PM assays, to test and compare >1000 bacterial species. The N-, P-, and S-source assays are not working for all these species, but the C-source and chemical sensitivity assays are. In terms of categorizing bacteria by the C-sources they consume and the inorganic ions they are or are not compatible with, the subset of PM1, PM2, and PM9 provides a useful and broadly applicable set of nearly 300 tests with which taxonomists can compare most or all fast-growing bacterial species. In fact, our research group has developed and recently released the new GEN III MicroPlate, which consists of our selection of the 94 best tests for bacterial species identification based on the set of 1920 PM tests (Franco-Buff *et al.*, 2008). This is the first time that a large number of both Gram-negative and Gram-positive bacteria could be phenotypically assayed and identified in a single, universal test panel.

The first new genus to be characterized with PMs and the GEN III MicroPlate is *Cronobacter*, formerly classified incompletely and incorrectly as *E. sakazakii. Cronobacter sakazakii* is an important pathogen in the infant formula industry because it has caused infant mortalities. The bacterium is often found in milk powders and is desiccation resistant. Iversen *et al.* (2007) have used both phenotyping and DNA analyses to describe and redefine *C. sakazakii* and four other named species: *Cronobacter malonaticus, Cronobacter muytjensii, Cronobacter turicensis*, and *Cronobacter dublinensis*. Interestingly, most clinically isolated strains in their collection were found to be dextrin positive and somewhat less salt tolerant, indicating possible pathoadaptation as these organisms move from environmental to clinical niches (Bochner *et al.*, 2008).

Schoolnik and colleagues (Keymer *et al.*, 2007) have used PM technology to help understand how environmental and ecological factors influence the genomic and phenotypic diversity of strains of the pathogen *V. cholerae*. Taking the technology in a different direction, Lenski, MacLean and colleagues have used PMs to simulate diverse culture environments with the objective of studying microbial adaption and evolution *in vitro*. The reader is directed to a number of interesting publications for more details (Cooper & Lenski, 2000; MacLean & Bell, 2002, 2003; Venail *et al.*, 2008). Yet another very different example is the work of Viti *et al.* (2007) to use PMs in a bioremediation context, to help understand how environmental strains of *Pseudomonas* adapt to becoming resistant to high levels of chromate.

## Uses in improving industrial bioprocesses

PM technology has numerous uses in industrial microbiology, particularly in efficiently optimizing the yields and improving the reproducibility of fermentation processes. For example, it can be used to characterize cell lines to select the best one to use in production, to understand in detail the culture properties of production cell lines, to understand how genetic changes affect production cell lines, to simulate hundreds/thousands of culture conditions, both the growth phase and production phase, to optimize culture conditions for both rapid growth and maximum product formation, and to test stock and inoculum cultures, as a quality control tool, to improve process consistency. With PM technology now available for bacterial, fungal, and animal cells, these advantages can be applied across a wide range of bioprocesses. The assays can be run kinetically and in high throughput to exploit the efficiencies that are inherent in the technology.

In terms of published examples, Druzhinina and colleagues (Druzhinina *et al.*, 2006; Seidl *et al.*, 2006; Nagy *et al.*, 2007) have used PM technology to understand and optimize cellulase, *N*-acetylglucosaminidase, and chitinase production by fungi, and Van Dyk *et al.* (2004) have used PM technology to understand and optimize the role of efflux pumps in enabling bacteria to tolerate higher levels of toxic end products. A general overview on bioprocess optimization has been written by Gorman (2003). Systems biology models are gaining increasing use and acceptance in industrial process development and this will undoubtedly lead to more integration of PM data to improve these models.

## Uses in systems biology

Systems biology seeks to model the cell as closely as possible and in its entirety (Ishii *et al.*, 2007). Modelers must rely on data available to them. However, information about the enzymes and pathways present in cells is much less comprehensive than generally acknowledged. Current definition and annotation of genes is rudimentary and still needs a lot of improvement, as evidenced by the data of Paulsen (Johnson *et al.*, 2008) evaluating transporter annotation and showing only 44% correct annotation. Understanding the regulation of genes in the context of the biology of the cell is an even much larger challenge. This is being addressed somewhat with mRNA measurements using genechips, but our understanding of gene regulation is still in its infancy. There are also major limitations to what can be concluded by mRNA measurements, because they do not tell us whether the transcribed gene is translated at a corresponding level and forms a protein that is active *in vivo*. We need *in vivo* biological measurements to provide that information, and PM is the most efficient technology for providing the first level of biological data.

In the last few years, there have been six publications on bacterial systems where Phenotype MicroArray data have been used to check on and improve systems models (Covert *et al.*, 2004; Feist *et al.*, 2007; Jones *et al.*, 2007; Mols *et al.*, 2007; Oh *et al.*, 2007; Oberhardt *et al.*, 2008). Of these, the publication by Oh and colleagues used PM data most comprehensively. The authors developed a method for reconciling growth profiling data with genome-scale models and used this method to greatly improve their models of *Bacillus subtilis*. Growth rates of this bacterium were computed with a predictive model and then compared with metabolic rate information from Phenotype MicroArray assays. Of 270 cases tested, the correct qualitative prediction rate was only 53%, but this improved to 79% after assimilating the phenotypic data and adding 84 reactions to the model. To test the new model further, predictions of the growth phenotypes of knockout strains were tested and found to be accurate in 725 of 772 (94%) cases. Overall the phenotypic data revealed the requirement for 89 specific enzymes that had not been annotated and the identification of 13 genes that could be putatively assigned to enzymes.

## Future directions of global phenotyping

PMs are a first attempt to provide a tool to globally analyze the biology of cell lines (phenomics). Just as with other ‘omics’ and related technologies (DNA sequencing, gene chips, proteomics, and metabolomics), the challenge is to expand the set of assays and make it more accurate, cheaper, easier, and robust, so that scientists can utilize the technology more often and more fully.

This can be accomplished, as it has with other technologies, by miniaturization. Current assays are run at a scale of 100 μL with about 10^6^ bacterial cells per well. Even a 4- to 10-fold miniaturization would give a substantial reduction in cost and increase in throughput. Although it is theoretically possible to go as low as a single cell per assay, one would probably never want to go that low because it would introduce noise due to the stochastic phenotypic behavior of individual cells. As long as there are 100 cells per assay or more, this should not be a problem.

The unifying theme of this journal issue is systems biology. PMs have been conceived of and designed as a technology to survey phenotypes of a cell from a physiological perspective. Physiology is a powerful approach to studying cells because it seeks to understand and enumerate the various subsystems that function within cells. Some of these are universal – necessary for all forms of life. Others are specialized functions found in specially differentiated cells and cells adapted to more unique environments. In my opinion, systems biology models would benefit greatly if they were more carefully and clearly organized around concepts of physiology and implemented with a physiological core structure.

Genomic maps and genomic annotation are driving systems biology. Although it is clear that annotation is evolving and needs improvement, there seems to be a comfort with and acceptance of genomic maps and the current modes of annotation. However, in 2003 (Bochner, 2003), I proposed the need for a second map, a phenotypic map, that would also be annotated in concert with genomic maps. To help bridge the gap between genomic maps and phenotypic maps, a realistic next step in the evolution of annotation is to annotate upstream regulatory sequences and gene regulation as thoroughly as protein-coding regions. If this can be done successfully it will take us to a new level of genome annotation and more importantly to a new level of understanding of biology. A first example in this direction is the recent publication (Jones *et al.*, 2007), in which phenotypically coregulated N-sources of *Pseudomonas fluorescens* were shown to share RpoN σ factor-dependent upstream binding sequences.
